# Effect of Culture at Low Oxygen Tension on the Expression of Heat Shock Proteins in a Panel of Melanoma Cell Lines

**DOI:** 10.1371/journal.pone.0037475

**Published:** 2012-06-22

**Authors:** Christopher Shipp, Evelyna Derhovanessian, Graham Pawelec

**Affiliations:** 1 Tübingen Ageing and Tumour Immunology Group, Department of Internal Medicine II, Centre for Medical Research, University of Tübingen, Tübingen, Baden-Württemberg, Germany; 2 Centre for Bioactive Discovery in Health and Ageing, University of New England, Armidale, New South Wales, Australia; The Moffitt Cancer Center & Research Institute, United States of America

## Abstract

**Objective:**

Tumours are commonly hypoxic and this can be associated with aggressive tumour type, metastasis and resistance to therapy. Heat shock proteins (hsps) are induced in response to hypoxia, provide cancer cells with protection against tumour-associated stressors and chaperone oncoproteins that drive tumour proliferation. This study examined the effect of different oxygen concentrations on the expression of hsps in melanoma cell lines.

**Methods:**

Melanoma cell lines were cultured in 2% and 20% O_2_. Expression of Hsp90, Hsp70, Hsp60, Hsp40 and Hsp32 proteins were determined by flow cytometry.

**Results:**

Growth rates and viability were reduced in the majority of cell lines by culture in 2% O_2_. Hsp expression was different in 2% compared to 20% O_2_ and changes in Hsp90 expression correlated with cell line generation time (P<0.005) and viability (P<0.01). Greater total hsp expression correlated with improved viability in 2% but not 20% O_2_ (P<0.05). Relative expression of the different hsps was consistent across cell lines and each correlated with the others (P = 0.0001) but not with Hsp32. Hsp expression was inversely correlated with cell line adhesion to laminin as well as collagen type IV and Breslow depth of the original primary tumour tissue (P<0.05), but not with Clark level or patient survival. All five hsps were identified on the cell surface.

**Conclusion:**

Culture in 2% O_2_ variably altered hsp expression in a panel of melanoma cell lines. Hsp expression was associated with certain cell line characteristics and clinical parameters of the originating tumour.

## Introduction

It is well established that hypoxia is a feature of human tumours [1], [2], [3]. The hypoxic state is the result of the combined effects of rapid proliferation of malignant cells and abnormal behaviour of blood vessels, resulting in insufficient blood supply to the tumour mass [1], [2], [3], [4]. Hypoxia contributes to the biology of tumours through multiple mechanisms including the promotion of genetic instability, contributing to immune evasion and assisting in the selection of cells more resistant to apoptosis and the harsh tumour microenvironment [2], [5], [6], [7], [8], [9]. Further, hypoxia has been associated with resistance to therapy, more aggressive tumours, tumour invasion, poor prognosis and patient death [5], [8], [10], [11]. Despite typically being a relatively small tumour frequently found on the skin, hypoxia is nevertheless a feature of human melanoma [1], [12], [13]. In melanoma, hypoxia is associated with tumour metastasis and may serve to enhance metastatic spread [14], [15], [16], [17].

The major mechanism by which cells respond to hypoxic stress is by rapid modulation of the expression of the HIF transcription factor [18]. HIF directly regulates the expression of heat shock proteins (hsps) and heat shock protein 90 (Hsp90) has been shown to assist in the stabilisation of HIF under hypoxic conditions in melanoma cells [19], [20]. Other studies suggest Hsp90 is involved in HIF expression and transactivation under hypoxia [21]. Hsps are an essential group of proteins that function as molecular chaperones and play a multitude of roles in eukaryotic cells [22]. Many of their functions contribute to the survival of tumour cells; accordingly, hsps have been shown to be abnormally expressed in a range of human cancers [23]. They promote the growth of cancer cells through multiple mechanisms such as inhibiting apoptosis, enhancing angiogenesis and providing protection against tumour-associated stressors such as hypoxia [23], [24], [25], [26], [27]. Hsps perform these roles in addition to chaperoning overexpressed oncoproteins that drive the proliferation of tumour cells [23], [28]. Consequently, hsps have been identified as valid targets in the treatment of cancer and are currently being evaluated in clinical trials in a number of cancer types including melanoma [29]. Hsp expression has been shown to be important in melanoma and relevant to patient clinical parameters such as Breslow depth, Clark level and survival [30], [31], [32], [33].

The relevance of hypoxia and hsps to cancer is well documented, but has rarely been studied in the context of human cancer cell cultures. Laboratory cell culture is routinely performed under hyperoxic conditions (i.e. in air) and this may be a limitation of this model for the study of human cancer cells which frequently experience hypoxia *in vivo*. Hsps have been investigated in melanoma tumour tissue and play multiple roles important for cancer growth, but have not been extensively studied in melanoma cell lines. Here, we present a preliminary study that sought to assess hsp expression in relation to melanoma cell line characteristics, patient clinical parameters and low and high oxygen culture conditions in a relatively large panel of melanoma cell lines.

**Figure 1:**
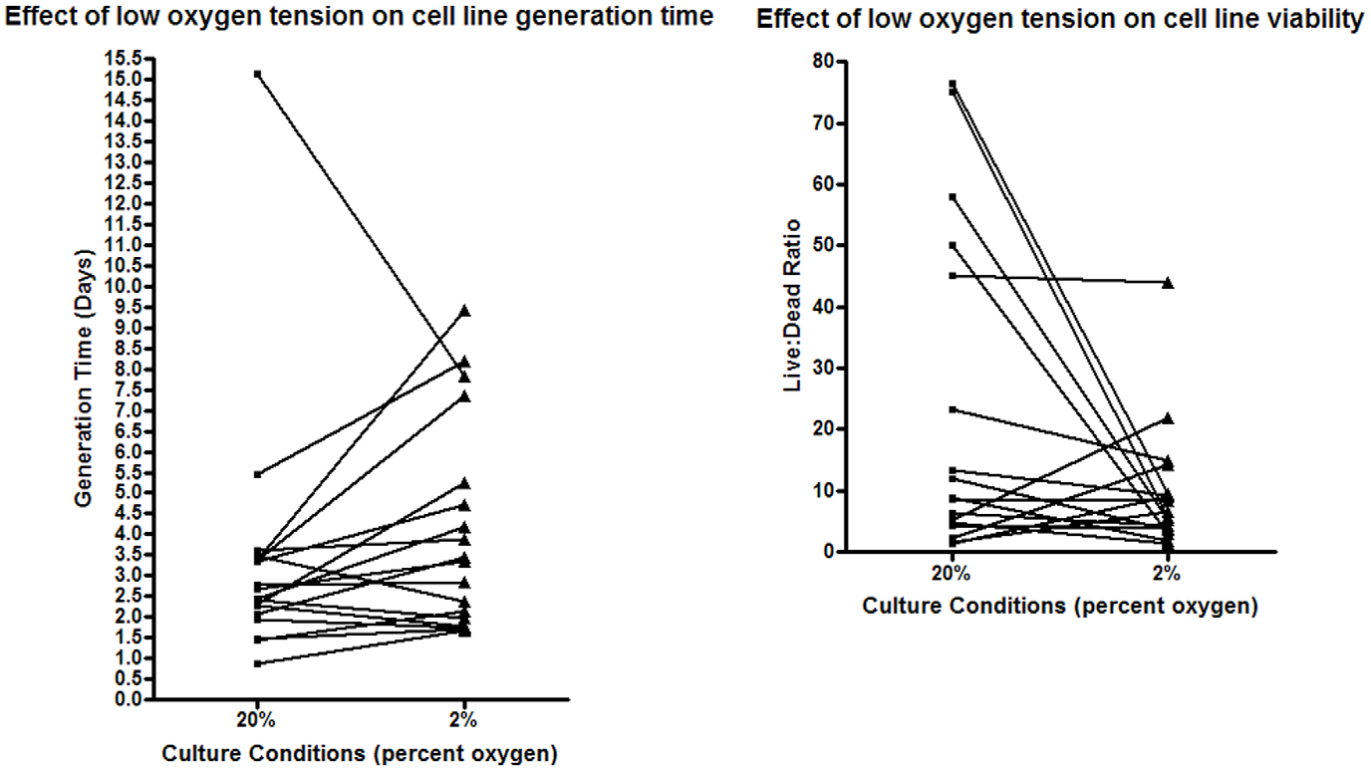
Effect of low oxygen culture on melanoma cell line generation time and viability. Eighteen melanoma cell lines were cultured under identical conditions in 2% and 20% O_2_ for five days. Following the culture period, the cell lines were harvested and living and dead cells enumerated using trypan blue. Total number of living cells was used to calculate generation time, and ratio of live to dead cells was used as a marker of cell viability.

## Methods

Cell lines: Melanoma cell lines were selected from the European Searchable Tumour Line Database (ESTDAB; http://www.medizin.uni-tuebingen.de/estdab/) which is the largest existing collection of melanoma cell lines. The ESTDAB cell lines have been thoroughly characterised and controlled with mycoplasma testing and DNA finger printing [34]. The sample cohort used in this study consisted of 40 cell lines of metastatic tumour origin and two of primary tumour origin (EST 66 and 83). For a full list of the cell lines used, see Fig. 3.

**Table 1:** Change in hsp expression in response to low oxygen tension.

Hsp	Decrease of hsp expressionin 2% O2 (% of cell lines)	Mean decrease (%)	Increase of hsp expressionin 2% O2 (% of cell lines)	Mean increase (%)
90	50.0	19.5	38.9	13.9
70	44.4	27.3	44.4	15.0
60	50.0	20.4	33.3	16.8
40	61.1	18.3	16.7	16.8

Eighteen melanoma cell lines were cultured under identical conditions in 2% and 20% O_2_. After five days the cell lines were harvested, stained with PE-conjugated hsp antibodies and protein expression assessed by flow cytometry. A Fluorescence Index was calculated (fold increase in mean fluorescence of the stained cells compared with the unstained cells) and was used as a comparative measure of protein expression.

**Table 2:** Association between change in Hsp90 expression and cell line generation time between 2% and 20% O_2_ culture.

	DecreasedHsp90 in 2%	IncreasedHsp90 in 2%
Decreased generation time in 2%	0	5
Increased generation time in 2%	8	2

P<0.005.

Changes in cell line generation time were correlated with changes in Hsp90 expression between the 2% and 20% O_2_ culture conditions.

**Figure 2:**
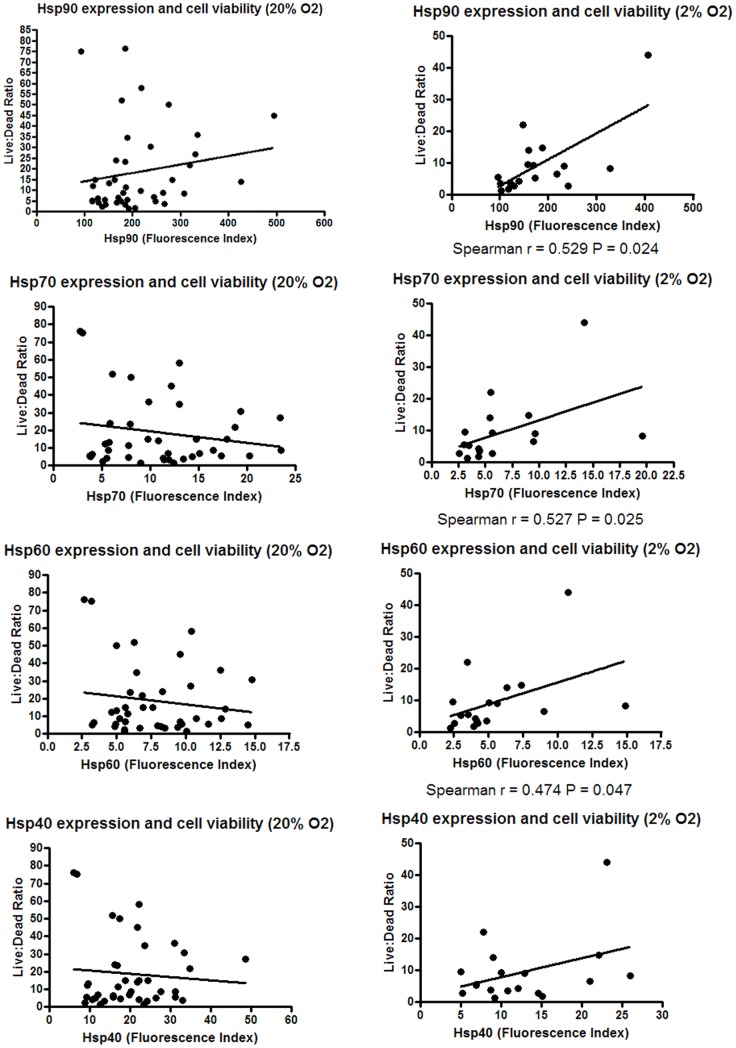
Effect of low oxygen culture on hsp expression. Forty two melanoma cell lines were cultured in 20% O_2_ and 18 of these cell lines additionally in 2% O_2_ for five days. Following the culture period, the cell lines were harvested and the number of living and dead cells determined using trypan blue. The ratio of live to dead cells was used as a marker of cell viability.

**Table 3:** Association between change in Hsp90 expression and cell line viability between 2% and 20% O_2_ culture.

	DecreasedHsp90 in 2%	IncreasedHsp90 in 2%
Decreased viability in 2%	7	2
Increased viability in 2%	0	4

P<0.01.

Changes in cell line viability were correlated with changes in Hsp90 expression between the 2% and 20% O_2_ culture conditions.

Cell culture: Cell lines were cultured with 35 ml RPMI 1640 medium (Life Technologies, Darmstadt, Germany) supplemented with 10% heat inactivated Foetal Calf Serum (FCS) (Sigma, Munich, Germany) in 75 cm^2^ cell culture flasks (Greiner Bio-One, Frickenhausen, Germany) for five days in an incubator (37°C, 5% CO_2_, 95% humidity) in air (20% O_2_) and in 2% O_2_ using the Concept 1000 Invivo_2_ (Ruskinn Technology, Pencoed, UK). Seeding cell number was adjusted according to the generation time (time required for one population doubling) of each cell line in order to avoid confluence and to obtain similar cell numbers at the end of the culture period. Following the culture period cells were washed with Hank's Buffered Salt Solution (HBSS) (PAA, Pasching, Austria) and detached from culture flasks by incubation with Trypsin-EDTA (Cambrex, Wiesbaden, Germany) (200 mg/l EDTA, 500 mg/l Trypsin 1∶250) for approximately three minutes. Trypsin was inactivated by adding of an equal volume of RPMI medium containing 10% FCS.

**Figure 3:**
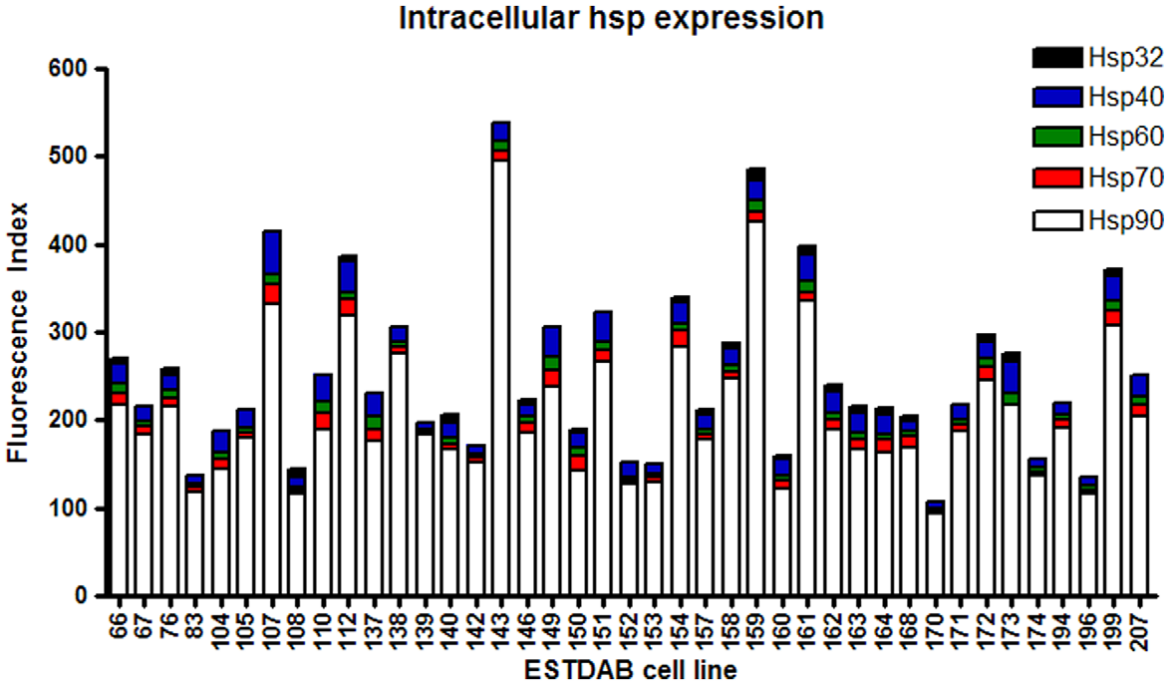
Hsp expression across melanoma cell lines. Forty two melanoma cell lines were cultured 20% O2 for five days. Following the culture period cell lines were harvested, stained with PE-conjugated antibodies to Hsps 90, 70, 60, 40 and 32 and protein expression assessed by flow cytometry. A Fluorescence Index was calculated (fold increase in mean fluorescence of the stained cells compared with the unstained cells) and was used as a comparative measure of protein expression across the cell lines.

Determination of cell number and viability: The trypan blue exclusion method was used to determine the number of living and dead cells. Melanoma cells were suspended in 10 ml of HBSS and mixed thoroughly. Equal volumes of a 0.4% trypan blue solution (Sigma) and the cell sample were mixed and applied to a Neubauer haemocytometer. Phase contrast microscopy was used to distinguish between stained (dead) and unstained (living) cells.

**Figure 4:**
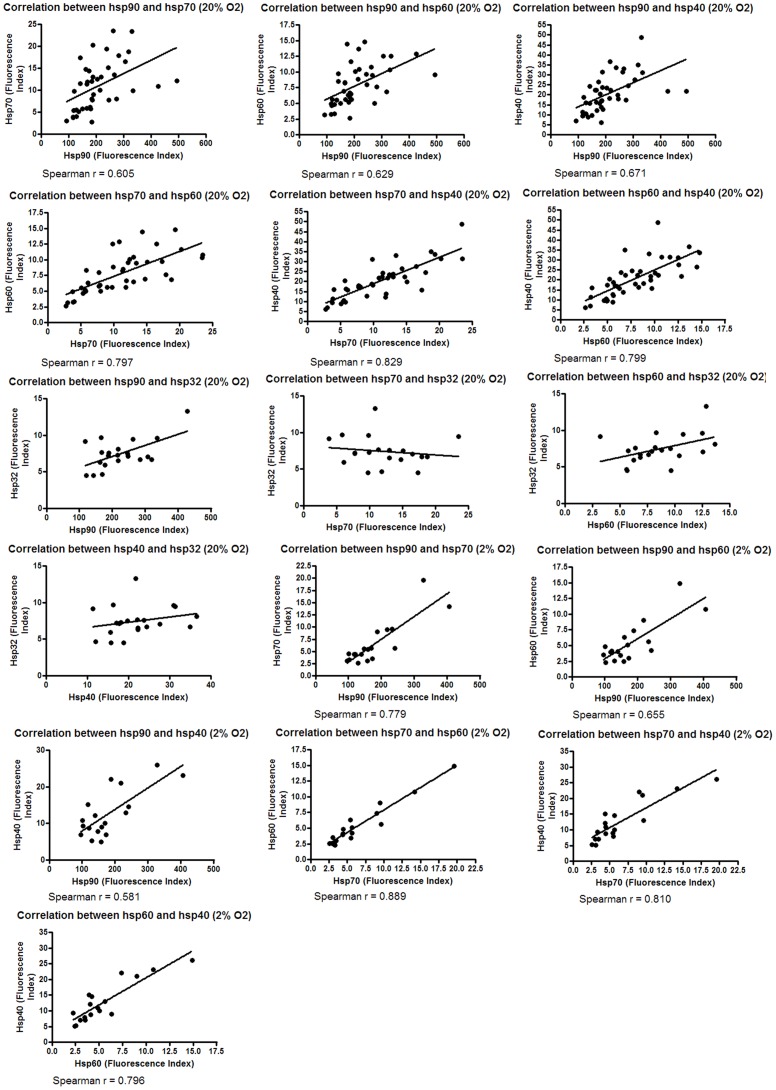
Correlation between the expression of different hsps. Melanoma cell lines were cultured in 2% O_2_ and 20% O_2_ for five days. Following the culture period cell lines were harvested, stained with PE-conjugated antibodies to Hsps 90, 70, 60, 40 and 32 and protein expression assessed by flow cytometry.

Flow cytometry: Following trypsinisation, 3.0×10^5^ cells were washed with PFEA (phosphate buffered saline, 2% FCS, 2 mM EDTA, and 0.01% azide) and Fc receptors blocked with GAMUNEX (Bayer, Leverkusen, Germany) on ice for ten minutes. Cells were washed with PFEA before being permeablised and fixed with Cytofix/Cytoperm and Perm/Wash (BD Biosciences, Heidelberg, Germany) according to the manufacturer’s instructions. Single fluorochrome staining was performed where antibody was diluted in Perm/Wash and incubated for 30 minutes on ice. PE-conjugated antibodies were used at the following concentrations: Hsp90 1:300 (product #SPA-830PE), Hsp70 1:100 (product #SPA-820PE), Hsp60 1:100 (product #SPA-807PE), Hsp40 1:100 (product #SPA-400PEF), Hsp32 1:100 (product #OSA-111PE) (all from Enzo Life Sciences, Lörrach, Germany). Following incubation cells were washed twice with Perm/Wash before being suspended in 120 µL of 1% formaldehyde solution (Merck, Darmstadt, Germany) in PFEA and stored on ice in a darkened container. For cell surface staining, PFEA was used in place of Cytofix/Cytoperm and Perm/Wash. Cells were immediately analysed on an LSR II flow cytometer with FACSDiva software (BD Biosciences). Data was analysed using FlowJo software (Tree Star, Ashland, USA). To perform data analysis, the main population of cells was gated on a forward scatter versus side scatter dot plot according to size and granularity. For each sample a fluorescence index (FI) was calculated in order to allow the comparison of fluorescence values for each cell line. The FI is the fold increase of the mean fluorescence intensity of the main population of cells in the stained cell sample by comparison with the corresponding unstained sample. FI values of less than two were considered negative.

**Figure 5:**
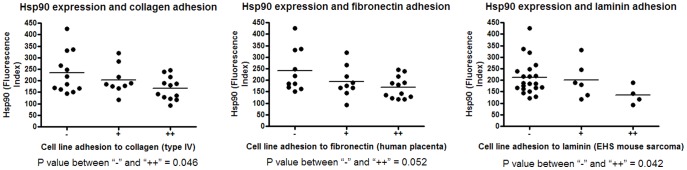
Correlation between Hsp90 expression and cell line ligand adhesion. Hsp expression was tested for correlation with the adhesion ability of these cell lines to the collagen type IV, fibronectin and laminin ligands.

Statistical analysis: Statistical analysis was performed using Prism software version 4.02 (GraphPad, San Diego, USA). Cell line ligand adhesion data was obtained from ESTDAB. Within matching data sets, changes of less than 5.0% were not considered to be different.

Paired two-tailed non-parametric t tests (Wilcoxon matched pairs tests) were used to assess significance between matching data points between two conditions.

Significance between two groups was assessed with two-tailed non-parametric (Mann-Whitney U) tests.

Correlations were assessed with non-parametric two-tailed (Spearman) correlation tests.

Trends across four grouping variables were assessed with two-tailed chi-squared contingency tests.

**Figure 6:**
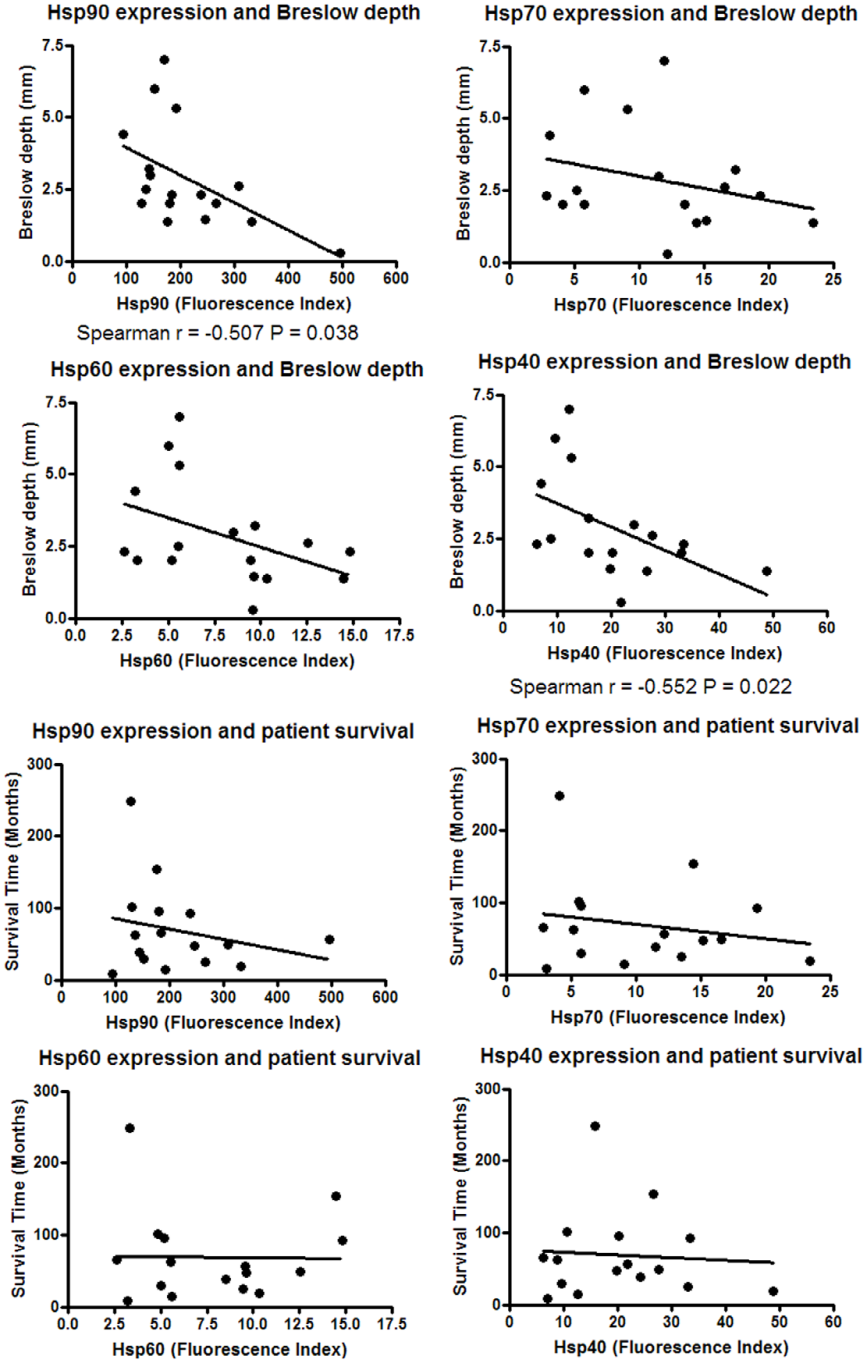
Relationship between hsp expression and patient clinical parameters. Hsp expression was investigated for association with melanoma patient clinical parameters.

## Results

### Low Oxygen Tension Reduces Viability and Retards Growth in the Majority of Melanoma Cell Lines

Eighteen melanoma cell lines were selected for culture in 2% O_2_. Twelve of 18 cell lines showed a mean increase of 70% in generation time when cultured in 2% O_2_ compared with the 20% O_2_ condition (P = 0.054). Five of the remaining six cell lines showed reduced generation times (mean decrease 26%), while the remaining cell line did not change (Fig. 1 A). Cell viability was accordingly reduced in 11 of 17 cell lines (P = 0.096), while four showed improved viability and two did not change (Fig. 1 B) (no data for 1 cell line). Four of the five cell lines that showed faster generation times in the 2% O_2_ condition also displayed improved viability in 2% O_2_ (EST 174, 194, 196 and 207), while one cell line showed no change for both generation time and viability (EST 143).

**Figure 7:**
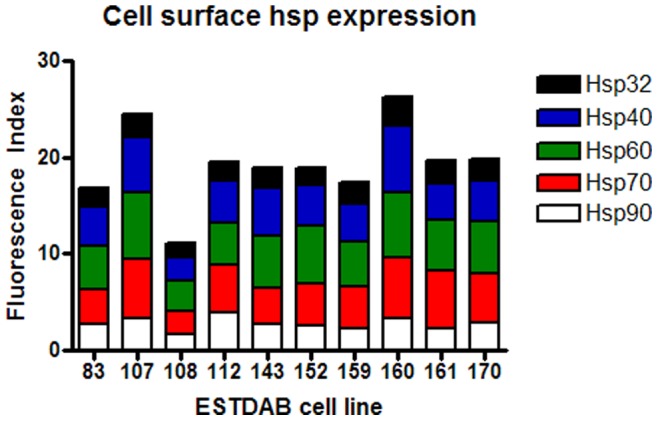
Cell surface expression of hsps on melanoma cell lines. Ten melanoma cell lines were cultured in 20% O_2_ for five days. Following the culture period cell lines were harvested, stained for cell surface Hsp90, Hsp70, Hsp60, Hsp40 and Hsp32 and protein expression assessed by flow cytometry. A Fluorescence Index was calculated (fold increase in mean fluorescence of the stained cells compared with the unstained cells) and was used as a comparative measure of protein expression.

### Low Oxygen Tension Results in Differential Heat Shock Protein Expression

Comparing hsp expression between the 2% and 20% O_2_ conditions showed that low oxygen culture resulted in up- or down-regulation of hsp expression (Table 1). For each of the four hsps examined, approximately half of the cell lines showed decreased expression in the 2% O_2_, while a smaller proportion showed increases. Six cell lines showed a reduction in the expression of all four hsps in the 2% O_2_ condition (EST 66, 76, 105, 108, 138 and 153), but the remaining cell lines up- and down-regulated these four hsps in response to low oxygen tension. Of note is that the four cell lines that showed faster generation times and improved viability in the 2% O_2_ condition showed a corresponding increase in Hsp90 expression in the 2% O_2_ culture condition.

### Heat shock Protein Expression is Associated with Melanoma Cell Line Generation Time and Viability

Because low oxygen tension resulted in altered hsp expression and influenced generation time and viability in these cell lines, the association of hsp expression with generation time and viability was investigated in the 2% and 20% O_2_ culture conditions. With the exception of Hsp60 in 20% O_2_ (P<0.03), total hsp expression was not associated with generation time in either condition (data not shown). When examining the increase or decrease in hsp expression in response to low oxygen tension, associations between the change in Hsp90 expression with cell line generation time (P<0.005, Table 2) were observed, but not for the other hsps (data not shown).

The relationship between hsp expression and viability in the 2% and 20% O_2_ culture conditions was then assessed. Significant relationships were found between higher expression of Hsp90, Hsp70 and Hsp60 and improved cell viability in the 2% (P<0.05) but not in the 20% O_2_ culture condition (Fig. 2). Examining the change in hsp expression between the 2% and 20% O_2_ culture conditions indicated that changes in Hsp90 expression were associated with cell line viability (P<0.01, Table 3).

### The Expression of the Different Heat Shock Proteins Correlates with one Another and with Melanoma Cell Line Adhesion

Hsp expression was compared throughout the cohort of melanoma cell lines cultured in 20% O_2_ (Fig. 3). Hsp90 was observed to account for the vast majority of total hsp expression in every cell line. The next most abundantly expressed hsps were Hsp40 and Hsp70, while Hsps 60 and 32 were similarly expressed at relatively low levels.

Given that the relative expression of hsps was observed to be consistent, relationships between the expression of these hsps were investigated. The expression of Hsp90, Hsp70, Hsp60 and Hsp40 was found to correlate, but not Hsp32 (in 2% (P = 0.015) and 20% O_2_ (P = 0.0001)) (Fig. 4, Hsp32 not tested in 2% O_2_).

Hsp90 is involved in cell adhesion [35] and for this reason a relationship between Hsp90 expression and adhesion to collagen type IV, fibronectin and laminin was investigated. An inverse correlation was found between the expression of Hsp90 and adhesion to these ligands (Fig. 5).

### Heat Shock Protein Expression Correlates with Breslow Depth but not Clark Level or Patient Survival

The expression of hsps in melanoma tissue has been shown to correlate with clinical parameters, but few, if any, studies have been performed on hsp expression in melanoma cell lines in this context. Patient survival time, Clark level and Breslow depth of the primary tumour tissue was known for a limited number of the metastasis-derived cell lines in the biobank used here, and were tested for a correlation with the expression of hsps. The expression of Hsp90 and Hsp40 was found to inversely correlate with Breslow depth, but no relationships were found with patient survival or Clark level (Fig. 6, Clark level data not shown).

### Hsps are Located on the Surface of Melanoma Cells as Well as Intracellularly

Cancer cells have previously been reported to express hsps on the cell surface and for this reason ten melanoma cell lines were screened for expression of cell surface hsps (Fig. 7). Hsps 90, 70, 60, 40 and 32 were observed to be expressed on all ten cell lines with the exception of three cell lines being negative for Hsp32 (EST 83, 108, 152) while one was negative for Hsp90 (EST 108). In comparison to intracellular expression, cell surface hsp expression was low. Of note is that Hsp90 was not the most highly expressed hsp on the cell surface, in contrast to its intracellular expression level. The expression of cell surface hsps did not correlate with intracellular hsp expression (P>0.35) (data not shown).

## Discussion

Cancer cell lines are widely used models in cancer studies, but standard practice dictates culture under hyperoxic conditions (i.e. in air). Since hypoxia is a common and important feature of cancer, culture of these cells under high oxygen conditions may contribute to the generation of misleading results. For this reason we investigated viability, generation time and hsp expression in order to assess melanoma cell line response to low oxygen tension. Although hypoxia is a feature of human melanoma, the majority of the cell lines tested here displayed retarded growth and reduced viability under low oxygen conditions. This may be due to the fact that they have been generated and cultured under hyperoxic conditions which have prevented the selection of hypoxia-resistant cells that would normally occur [36]. Hypoxia is a known inducer of the hsp response, but to the best our knowledge, no studies have examined the effect of low oxygen on hsp expression in melanoma or indeed in any other cancer cell lines in the manner performed here [37]. Unexpectedly, a widespread induction of hsp expression did not occur in response to low oxygen tension. Despite this, changes in Hsp90 expression were associated with tolerance to low oxygen as measured by generation time and viability. These data suggest that improved hypoxic tolerance is associated with the induction of hsps in melanoma cells. Further, higher total hsp expression was associated with improved viability in low but not high oxygen conditions, perhaps reflecting the anti-apoptotic role that hsps play in cancer [24], [38]. The greater demand placed on the hsp chaperone system under low oxygen stress when the level of hsp expression is more likely to be below the threshold required for cellular survival may account for the observation that hsp expression was not associated with viability under high oxygen levels. If these observations are at all reflective of *in vivo* conditions then it follows that anti-neoplastic hsp inhibiting drugs may be relatively more effective in hypoxic tumours in which a range of standard therapies are known to be less effective [5]. Indeed, previous reports have shown that melanoma cells require hsps in order to proliferate [39], [40]. Thus, *in vivo* under hypoxic conditions their effectiveness may be enhanced. Models that allow regions of variable oxygen concentration, such as three dimensional culture models or *in vivo* xenografts, could be used to confirm or refute the results presented in this study.

Relative hsp expression was observed to be consistent across the cohort of cell lines and levels of Hsp 90, 70, 60 and 40 expression were shown to correlate with one another but not with Hsp32. Hsp90 is essential for eukaryotic cell viability and it chaperones a large number of overexpressed client proteins in cancer. This may explain why Hsp90 was expressed at a level many fold greater than the other hsps examined [22], [41]. However hsps most often function in coordination with other molecular chaperones [22], [42]. The other hsps examined in this study are major Hsp90 co-chaperones, consistent with the notion that hsps cooperate closely in their role as molecular chaperones [22]. The intimacy of their association may explain the observation that their expression correlated with one another but not with Hsp32, because this protein is not a molecular chaperone and its function is less related to that of the other hsps [43].

Hsp expression was observed to correlate with Breslow depth but not Clark level or patient survival. Studies using primary melanoma tissue have shown increased Hsp90 expression to be associated with greater Breslow depth and Clark level [33], but in the present study with metastatic melanoma cell lines an inverse relationship was observed. This relationship was observed even though these lines have been cultured for several generations under *in vitro* conditions. Similar reports exist in other contexts [44]. For example, Walter *et al*. demonstrated MMP-2 mRNA levels in renal cancer cell lines to inversely correlate with patient survival [45]. A potential disadvantage of using cultured cells as models for *in vivo* processes is that it is not known how reflective the parameters measured under these conditions are of *in vivo* conditions. The data presented here coupled with at least one previous report may suggest that cancer cell lines maintain a degree of relevance to patient clinical parameters and thus may be of use as models for cancer. This relevance may be maintained indirectly, since results from previous studies using melanoma tissue suggest that in contrast to the results of this study using cell lines, that Breslow depth is positively associated with hsp expression. Since in these cell lines hsp expression was relevant to viability in 2% but not 20% O_2_, culture under low oxygen tension may be more relevant to *in vivo* growth conditions, but further studies are needed to confirm these preliminary findings. Of note is that the inverse relationship between hsp expression and Breslow depth was observed in 20% O_2_, and it is not known if hsp expression in 2% O_2_ also correlates with Breslow depth. No remarkable differences were observed between the primary-derived and metastasis-derived cell lines in this study.

It should be noted that the observation of an inverse correlation between hsp expression and Breslow depth does not imply that this relationship is present in tumours *in vivo*, as the level of hsp expression observed in these cell lines may not be reflective of the level *in vivo*. This could be due to the influence of the artificial cell culture environment. Follow-up studies in tumours and cell lines will confirm or refute the preliminary findings presented here. If this observation is confirmed, further studies could examine other clinical and biological features such as the expression of inflammatory cytokines, tumour ulceration, regional differences in the distribution of hsp expression (for example, proximity to the invasive front, necrotic core or blood vessels), histological sub-type or other factors that may interact with or separately explain this observation. If the level of hsp expression observed in this study is reflective of *in vivo* expression, then studies using melanoma tissue that have shown higher hsp expression to be associated with greater Breslow depth [33] contradicts the observations here that higher hsp expression correlates with reduced Breslow depth. This contradiction might be explained by hsps influencing the immunogenicity of these cells, producing a more effective immune response and a smaller resultant tumour [46], [47]. Supporting this is a study in which expression of Hsp70 was found to correlate with improved survival in melanoma patients [31]. Further, immune recognition is a potential function for the hsps identified on the cell surface as membrane-located hsps have been shown to act as targets for T cells and NK cells [48], [49]. The finding here that Hsp90 and Hsp70 are expressed on the surface of melanoma cells confirms previous studies, but to the best of our knowledge the expression of Hsp60, Hsp40 or Hsp32 has not previously been reported [50], [51].

Although this investigation is not an extensive study into the effects of low oxygen tension on cancer cell cultures, it does present novel findings with respect to the potential role of hsps and demonstrates that further investigation into the effects of oxygen tension on cancer cell cultures is warranted. These data suggest that hsps associate closely in melanoma cells and that their expression is associated with tolerance to low oxygen tension and patient clinical parameters. The *in vivo* importance of hypoxia coupled with the finding that hsp expression is relevant to melanoma cell viability in low but not high oxygen tension suggests cancer cell cultures should more frequently be subjected to oxygen tension that more closely resembles *in vivo* levels in order to cast more light on the largely unknown influence that this condition has on the myriad of facets investigated in modern cancer research.
